# Swimming exercise ameliorates insulin resistance and nonalcoholic fatty liver by negatively regulating PPARγ transcriptional network in mice fed high fat diet

**DOI:** 10.1186/s10020-023-00740-4

**Published:** 2023-10-31

**Authors:** Yong Zhang, Jie Xu, Di Zhou, Tingting Ye, Puqing Zhou, Zuofeng Liu, Xinyuan Liu, Zinan Wang, Tianmiao Hua, Zhenghao Zhang, Qingyan Sun

**Affiliations:** 1 Physiology laboratory of College of Life Sciences, Anhui Normal University, Wuhu, China; 2 the State Key Laboratory of Cellular Stress Biology, Innovation Center for Cell Biology, School of Life Sciences, Xiamen University, Xiamen, China; 3 Department of Hepatology, Affiliated Hospital of Panzhihua University, Panzhihua, China; 4 Department of Hematology, People’s Hospital of Xinjiang Uygur Autonomous Region, Urumqi, China

**Keywords:** Swimming exercise, Insulin resistance, Non-alcoholic fatty liver disease, PPARγ signaling, Lipid metabolism

## Abstract

**Background:**

Recent findings elucidated hepatic PPARγ functions as a steatogenic-inducer gene that activates *de novo* lipogenesis, and is involved in regulation of glucose homeostasis, lipid accumulation, and inflammation response. This study delved into a comprehensive analysis of how PPARγ signaling affects the exercise-induced improvement of insulin resistance (IR) and non-alcoholic fatty liver disease (NAFLD), along with its underlying mechanism.

**Methods:**

Chronic and acute swimming exercise intervention were conducted in each group mice. IR status was assessed by GTT and ITT assays. Serum inflammatory cytokines were detected by Elisa assays. PPARγ and its target genes expression were detected by qPCR assay. Relative protein levels were quantified via Western blotting. ChIP-qPCR assays were used to detect the enrichment of PPARγ on its target genes promoter.

**Results:**

Through an exploration of a high-fat diet (HFD)-induced IR and NAFLD model, both chronic and acute swimming exercise training led to significant reductions in body weight and visceral fat mass, as well as hepatic lipid accumulation. The exercise interventions also demonstrated a significant amelioration in IR and the inflammatory response. Meanwhile, swimming exercise significantly inhibited PPARγ and its target genes expression induced by HFD, containing CD36, SCD1 and PLIN2. Furthermore, swimming exercise presented significant modulation on regulatory factors of PPARγ expression and transcriptional activity.

**Conclusion:**

The findings suggest that swimming exercise can improve lipid metabolism in IR and NAFLD, possibly through PPARγ signaling in the liver of mice.

**Supplementary Information:**

The online version contains supplementary material available at 10.1186/s10020-023-00740-4.

## Introduction

Type 2 diabetes mellitus (T2DM) and nonalcoholic fatty liver disease (NAFLD) are prevalent metabolic disorders that contribute to a destructive cycle of complications. This cycle accelerates the escalation of vascular complications related to T2DM and exacerbates the progression of NAFLD to liver cirrhosis and even hepatocellular carcinoma. Insulin resistance (IR) is a critical contributor to the pathogenesis of T2DM and other metabolic disorders(Choi and Jung 2018). IR is widely believed to be the primary independent risk factor for NAFLD(Targher et al. 2016; Park et al. 2004). (Gao et al. 2010; Liu et al. 2014a, b). The development of insulin resistance (IR) occurs at a molecular level when there is an imbalance between excessive nutrients or inflammatory cytokines and the cell membrane receptors(Lonardo et al. 2015; Boden 2011), which IRS1 phosphorylation at serine307 and sequentially results in compromised ability of IRS1 to combine with insulin receptors and then decreases Akt phosphorylation at serine473, and then resulted in the inhibited potency of insulin signal transduction. This in turn inhibits glycogen synthesis by stimulating gluconeogenesis through elevating the phosphoenolpyruvate carboxykinase (PEPCK) and glucose-6-phosphatase (G6Pase) protein levels(Liu et al. 2014a, b).

On the other hand, NAFLD is a medical condition characterized by the accumulation of an excessive amount of fat in the liver, attributed to factors other than alcohol consumption (Li et al. 2014). Currently, NAFLD is the most prevalent type of chronic liver disease globally, and it affects nearly 70% of individuals who are overweight (Wei et al. 2016). Researches have demonstrated that NAFLD is not only associated with morbidity and mortality related to the liver, but it also elevates the risk of developing diabetes and cardiovascular diseases(Meex and Watt 2017; Lavoie and Pighon 2012; Sun et al. 2015; Luo et al. 2015). This chronic liver condition exacerbates in conjunction with IR, often remaining asymptomatic until the patient develops cirrhosis (Xue et al. 2022). Therefore, it is essential to take preventive measures and address IR and NAFLD at the early stages of their development.

Abnormalities in fat metabolism are associated with NAFLD. Peroxisome proliferator-activated receptor γ (PPARγ) is a member of the nuclear hormone receptor superfamily, which has been found to participate in the regulation of glucose homeostasis(Xu et al. 2021), adipogenesis(Garin-Shkolnik et al. 2014), and inflammation(Luo et al. 2022). Many studies have demonstrated that hepatic PPARγ serves as a gene that promotes fat accumulation by stimulating the *de novo* lipogenesis(Ning et al. 2022; Kim et al. 2017; Tong et al. 2019), containing fatty acid translocase (FAT/CD36), stearoyl-CoA desaturase 1 (SCD1), and perilipin 2 (PLIN2), and increases hepatic triglyceride accumulation(Matsusue et al. 2008; Zhou et al. 2012). PPARγ expression and transcriptional activity were regulated by several factors. Interferon regulatory factor 6 (IRF6) and hepatocyte nuclear factor 1α (HNF1α) both function as transcription factor, which binds directly to the promoter region of the PPARγ gene, thereby inhibiting the transcription of both PPARγ and its target genes responsible for regulating lipogenesis and fatty acid uptake (Tong et al. 2019; Patitucci et al. 2017). Peroxisome proliferator-activated receptor (PPAR)-γ coactivator 1α (PGC-1α), belongs to a small family of transcriptional coactivators, that includes the close homolog PGC-1β and PGC-1-related coactivator, which is the well-known coactivator for PPARγ functions as transcription factor (Puigserver and Spiegelman 2003). Furthermore, the retinoic acid receptor-related orphan receptor-α (RORα regulates hepatic lipid homeostasis by exerting a negative effect on the transcriptional activity of PPARγ via specifically binding and histone deacetylase 3 (HDAC3) to the promoters of PPARγ target genes, leading to transcriptional repression of PPARγ (Kim et al. 2017).

Due to insulin resistance being the primary pathogenic factor in NAFLD, drugs aimed at targeting insulin resistance have been utilized in the treatment of NAFLD. Various thiazolidinediones, which are primarily utilized for improving insulin resistance and treating type 2 diabetes, have been demonstrated efficacy in mitigating hepatic steatosis and fibrosis among individuals with NAFLD. Despite the prevalence of NAFLD, there are currently limited pharmacological treatments available that have been validated for this condition (AISF 2017; Lonardo et al. 2017). The current approach to managing NAFLD involves lifestyle changes, weight loss, and exercise. Exercise is a safe and effective way to improve both insulin resistance and NAFLD(Yu et al. 2016; Romero-Gómez et al. 2017), although the exact mechanisms involved are not yet fully understood.

In the present study, we investigated the hypothesis that exercise might ameliorate IR and NAFLD through regulating PPARγ transcriptional network. In order to achieve this objective, we established a mouse model of IR and NAFLD via high fat diet (HFD) feeding. Next, we investigated the underlying mechanism through which exercise impacts IR and NAFLD by analyzing the expression of PPARγ and its target genes in the livers of mice fed a high-fat diet.

## Materials and methods

### Experimental animals and grouping

Forty-eight male C57/BL6 mice, aged 7 weeks and weighing between 20 and 22 g, were acquired from Nanjing Qing-Long-Shan Animal Breeding Farm (Nanjing, China, Certificate: No. SX1207). All the mice were kept in a controlled environment with a temperature maintained at 25 ± 1℃ and a humidity level of approximately 55%, following a 12-hour light/dark cycle. They were provided with unlimited access to chow and sterile water. All experimental protocols were reviewed and authorized by the local animal ethics committee (Anhui Normal University, Wuhu, China). One week after feeding adaptation, the mice were randomly divided into normal control (NC) group, diet-induced obesity sedentary (DIO-SED) group, diet-induced obesity plus chronic exercise (DIO-CE) group, and diet-induced obesity plus acute exercise (DIO-AE) group (n = 12 per group). the NC group was fed a normal diet, the other groups were all fed a 60% kcal fat HFD for 16 weeks.

### Chronic and acute exercise protocol

Weight-loaded swimming training was performed in this study, as we previously described(Zhang et al. 2019, 2021). In briefly, the mice in the DIO-CE group underwent an 8-week swimming training program with a 5% load of their body weight. Similarly, the DIO-AE group mice participated in two 3-hour swimming sessions under identical conditions at the 17th week. Meanwhile, the NC and DIO-SED group mice were kept in the same experimental conditions, but the water level was adjusted to minimize any stress caused by the water.

### Glucose tolerance test (GTT) and insulin tolerance test (ITT)

For the evaluation of glucose tolerance and insulin tolerance, mice of each group were divided into two groups(n = 6), and then separately were intraperitoneal injection of glucose (1 g/kg BW) or insulin (0.75U/kg, Novolin R, Novo Nordisk, Denmark) after overnight fasting. Blood glucose levels were detected from the tail at baseline and 15, 30, 60, 90 and 120 min after injection using a glucose meter (Roche, Germany).

### Measurements of metabolic indices and liver lipid analyses

To minimize the immediate impact of exercise, mice in the DIO-CE group were anesthetized 48 h after their last swimming session. The DIO-AE group fasted for 12 h after their final training and were then administered 10% chloral hydrate via intraperitoneal injection to induce anesthesia 16 h later. Blood samples were obtained from the orbit and the serum was separated and stored for the purpose of detecting blood biochemical markers.

The fasting blood glucose levels, fasting serum insulin levels, and homeostatic model assessment of insulin resistance (HOMA-IR) values were evaluated as previously described(Luo et al. 2018; Zhao et al. 2018). Briefly, The HOMA-IR index was computed using the subsequent equations: HOMA-IR = (FBG ×FINS)/22.5. Here, FBG represents the fasting blood glucose level and FINS represents the fasting insulin level. To determine the glycogen content of the tissues, glycogen assay kit (Solarbio, China) was used to determine the content of glycogen in liver fragments.

Liver and serum triglyceride (TG) levels, total cholesterol (TC) levels, non-esterified fatty acid (NEFA) levels were detected via biochemical kits (Nanjing Jiancheng, China) follow manufacturer’s instruction. The determination of glycogen content in liver fragments was carried out using the Liver Glycogen Assay Kit (Solarbio, China), following the manufacturer’s instructions.

### Inflammatory cytokines analyses

The serum cytokines levels including TNFα and IL-1β were detected via Elisa kit (Boster, China) follow manufacturer’s instruction.

### Histological analysis

The liver specimens were embedded in paraffin and subsequently stained with hematoxylin and eosin (H&E) for the purpose of visualizing the lipid accumulation pattern. To visualize the accumulation of lipid droplets in the liver, frozen liver sections were prepared in Tissue-Tek OCT compound and stained with Oil Red O.

### Liver function assay

The liver function was assessed by measuring the levels of alanine aminotransferase (ALT) and aspartate aminotransferase (AST) in serum, utilizing biochemical assay kits (Nanjing Jiancheng, China).

### Total RNA isolation and quantitative RT-PCR assay

Total RNA was extracted from liver tissues using TRIzol reagent (Abclonal, Wuhan, China), and the reverse transcription was performed on a 2 µg of total RNA using a FastQuant RT Kit (with gDNase) (Tiangen Biotech, Beijing, China) following the manufacturer’s instructions. RT-PCR was performed using SYBR Green PCR Master Mix (Abclonal, Wuhan, China) on Bio-Rad CFX Manager. The mRNA levels of genes related to inflammation and fatty acid metabolism were normalized using β-actin as the housekeeping gene. The primer pairs utilized in this study are listed in the Supplementary Information.

### Tissue extraction and immunoblotting analysis

Tissue extraction and Immunoblotting were performed as previously described(Zhang et al. 2019, 2021). Briefly, liver tissues were lysed in ice-cold RIPA lysis buffer, supplemented with protease and phosphatase inhibitors. The BCA protein assay kit was used to measure protein concentrations. Protein samples (30 µg) was separated on 8-12% SDS-PAGE gels and transferred to PVDF membranes (Millipore, USA). After being blocked with 5% skim milk, the membranes were subjected to overnight incubation with primary antibodies at 4℃. This was followed by a 1-hour incubation with the appropriate secondary antibodies at room temperature. A chemiluminescence system (Tianneng, China) was employed to visualize the results. The bands intensities were quantified with Image Pro Plus software and normalized to the loading control β-actin. The antibody details are listed in Supplementary information.

### Chromatin immunoprecipitation (ChIP) assays

The ChIP assays were conducted as described(Kim et al. 2017). The livers of mice were collected and washed with PBS before being crosslinked with 1% formaldehyde for 10 min at room temperature. The reaction was then halted by treating the livers with a 0.125 M glycine solution for 5 min. Subsequently, the mouse livers were washed twice with ice-cold PBS. DNA was purified with a Sonication ChIP Kit (RK20258, Abclonal, China). The DNA that was precipitated underwent quantitative PCR analysis. Real-time quantitative PCR was performed using 2 µl of the 100 µl DNA extractions, with all reactions done in triplicates. Supplementary information lists the primers used for the analysis.

### Statistical analysis

The data for each group were presented as mean ± SD. To compare the statistical differences between groups, the t-test and one-way analysis of variance were utilized. All statistical analyses were conducted through GraphPad Prism 8.0 software, with a significance level of *P* < 0.05.

## Results

### Swimming exercise ameliorates obesity-induced insulin resistance and hepatic insulin signaling transduction

**Fig. 1:**
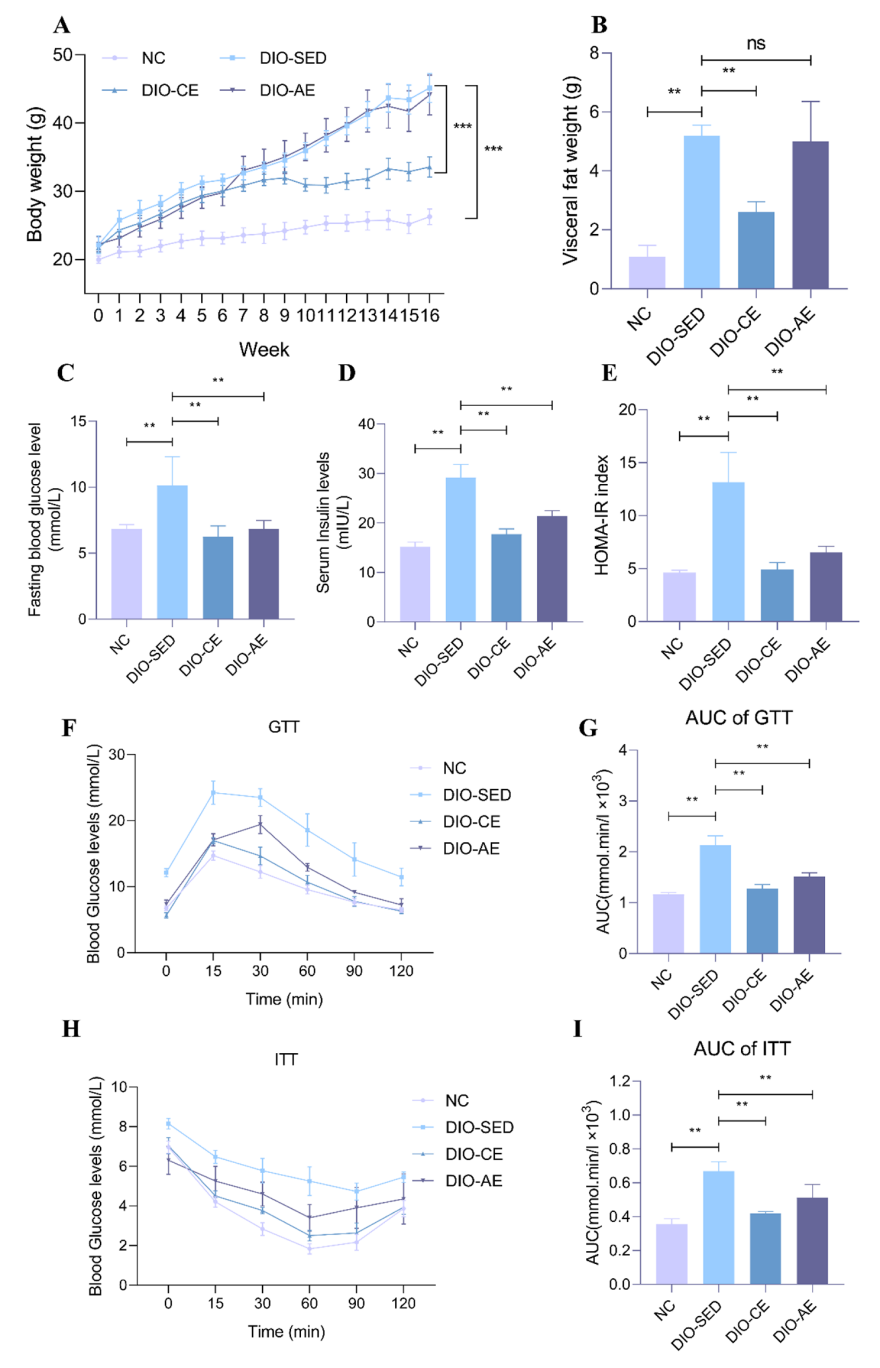
Swimming exercise ameliorates insulin resistance. **A** Body weight, **B** Visceral fat weight, **C** Fasting blood glucose levels, **D** Fasting insulin levels, **E** HOMA-IR index, **F** Glucose tolerance test (GTT) and **G** the corresponding area under the curve (AUC), **H** Insulin tolerance test (ITT) and **I** the corresponding area under the curve (AUC). Data is presented as mean ± SD. One-way ANOVA was used to determine a significant difference between groups. *p < 0.05, **p < 0.01 between groups

Throughout the course of the experiment, the body weight of the mice was measured on a weekly basis. Compared with the NC group mice, the DIO-SED group presented significantly higher body weight and visceral fat mass. After an 8-week swimming training program, there was a significant reduction in body weight and visceral fat mass. However, acute swimming training did not result in a significant reduction in these two parameters. (Fig. 1A, B).

**Fig. 2:**
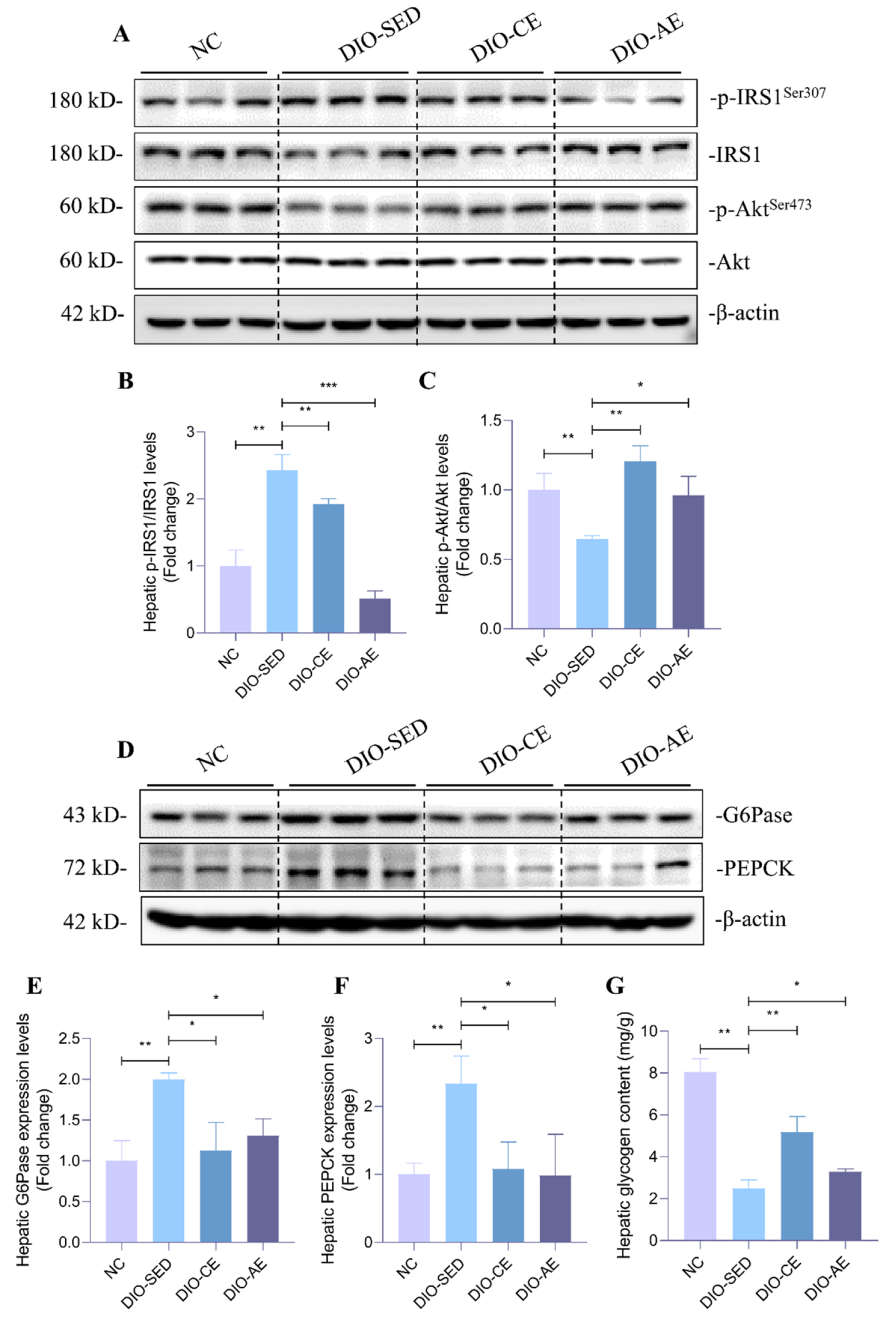
Swimming exercise enhances insulin signaling and inhibits gluconeogenesis. **A-C** Western blotting of IRS1/AKT in liver of mice and corresponding quantitative analysis, **D-F** Western blotting of G6Pase and PEPCK in liver of mice and corresponding quantitative analysis. **G** Glycogen contents in liver of mice. Data is presented as mean ± SD. n = 6. All experiments were repeated at least 3 times independently. A two-tailed Student’s *t*-test was performed for comparison of results between indicated groups. *p < 0.05, **p < 0.01 between groups

To investigate the effects of swimming exercise on glucose metabolism in IR mice, we measured their fasting blood glucose levels (FBG), fasting insulin levels (FINS), and the homeostatic model assessment of insulin resistance (HOMA-IR) index. The results showed that DIO-SED mice exhibited higher FBG, FBI and HOMR-IR indexes than NC mice (Fig. 1C-E). In addition, the glucose tolerance and insulin sensitivity of DIO-SED mice were found to be worsened when compared to NC mice (Fig. 1F-I). Compared with DIO-SED mice, FBG, FINS and HOMA-IR exhibited significant reduction (Fig. 1C-E), moreover, glucose tolerance and insulin sensitivity were largely ameliorated in DIO-CE and DIO-AE mice (Fig. 1F-I). The reduction in insulin sensitivity is primarily caused by disruptions in insulin signaling transduction(Zhao et al. 2018). To assess this, we conducted western blot analysis to investigate the IRS1/Akt signaling pathway, and the results demonstrated that p-IRS1^Ser307^ was increased, while p-Akt^Ser473^ was decreased in DIO-SED mice compared with NC mice. Conversely, p-IRS1^Ser307^ was decreased, while p-Akt ^Ser473^ was increased in DIO-CE and DIO-AE mice. (Fig. 2A-C). Moreover, the protein levels of gluconeogenesis-related genes (PEPCK and G6Pase) were higher in DIO-SED mice than in NC mice. Compared with DIO-SED mice, PEPCK and G6Pase protein levels were significantly decreased in DIO-CE and DIO-AE mice (Fig. 2D-F). As a result, the glycogen levels in the livers of DIO-SED mice were mostly maintained, while there was a significant increase in glycogen content in the livers of both DIO-CE and DIO-AE mice (Fig. 2G). Collectively, these results indicates that swimming exercise ameliorates HFD-induced insulin resistance.

### Swimming exercise ameliorates HFD-induced hepatic steatosis and inflammation response

**Fig. 3:**
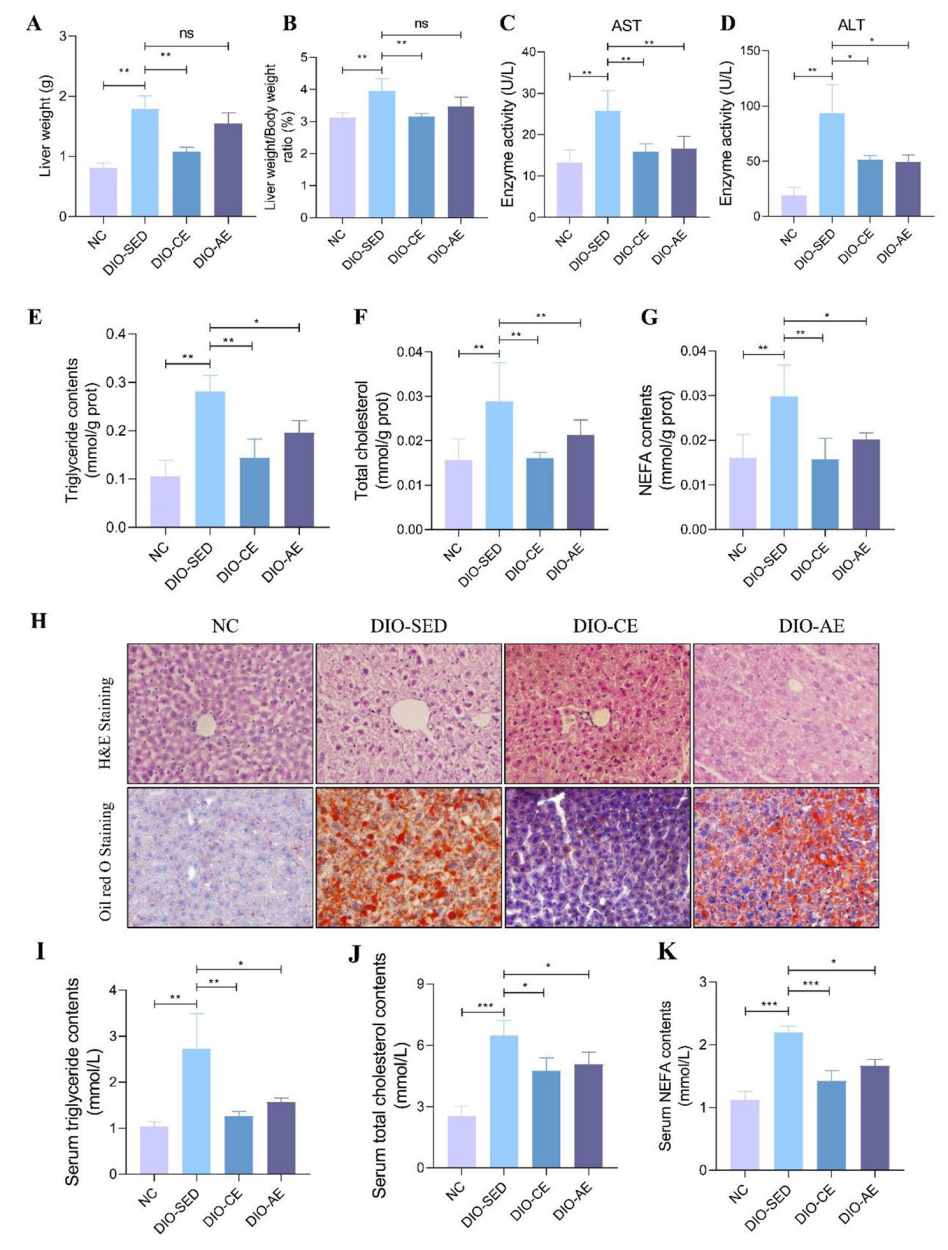
Swimming exercise ameliorates HFD-induced hepatic steatosis. **A** Liver weight, **B** Liver weight/body weight ratio, **C** the levels of AST and **D** ALT in the indicated groups. **E** triglycerides (TG) contents, **F** total cholesterol and **G** NEFA contents in liver of the indicated groups. **H** H&E and Oil Red O staining of liver sections of mice. **I** triglycerides (TG) contents, **J** total cholesterol and **K** NEFA contents in serum of the indicated groups. Data is presented as mean ± SD. One-way ANOVA was used to determine a significant difference between groups. *p < 0.05, **p < 0.01 between groups

Hepatic steatosis is a significant characteristic of NAFLD. This condition can be further worsened by insulin resistance, and is often caused by prolonged consumption of HFD (Tilg and Moschen 2008). The findings of the study revealed that DIO-SED mice had higher liver weight and liver weight to body weight (LW/BW) ratios compared to NC mice (Fig. 3A, B). Furthermore, the levels of TG, TC, and NEFA were significantly elevated in the livers of DIO-SED mice when compared to NC mice (Fig. 3E-G). These results were further corroborated by H&E and Oil Red O staining, which showed that HFD treatment led to greater lipid accumulation in the livers of DIO-SED mice than in those of NC mice (Fig. 3H). Conversely, liver weight and LW/BW ratio showed significantly decreased in DIO-CE mice, rather than DIO-AE mice compared with DIO-SED mice (Fig. 3A, B). And the TG, TC and NEFA levels were decreased in liver of DIO-CE and DIO-AE mice compared with DIO-SED mice. H&E and Oil Red O staining revealed a reduction in lipid accumulation in the livers of DIO-CE and DIO-AE mice when compared to DIO-SED mice (Fig. 3E-G). Additionally, the serum enzyme activities of ALT and AST were observed to be significantly higher in DIO-SED mice when compared to NC mice. However, a significant decrease in enzyme activity of ALT and AST was observed in DIO-CE and DIO-AE mice when compared to DIO-SED mice (Fig. 3C, D).

**Fig. 4:**
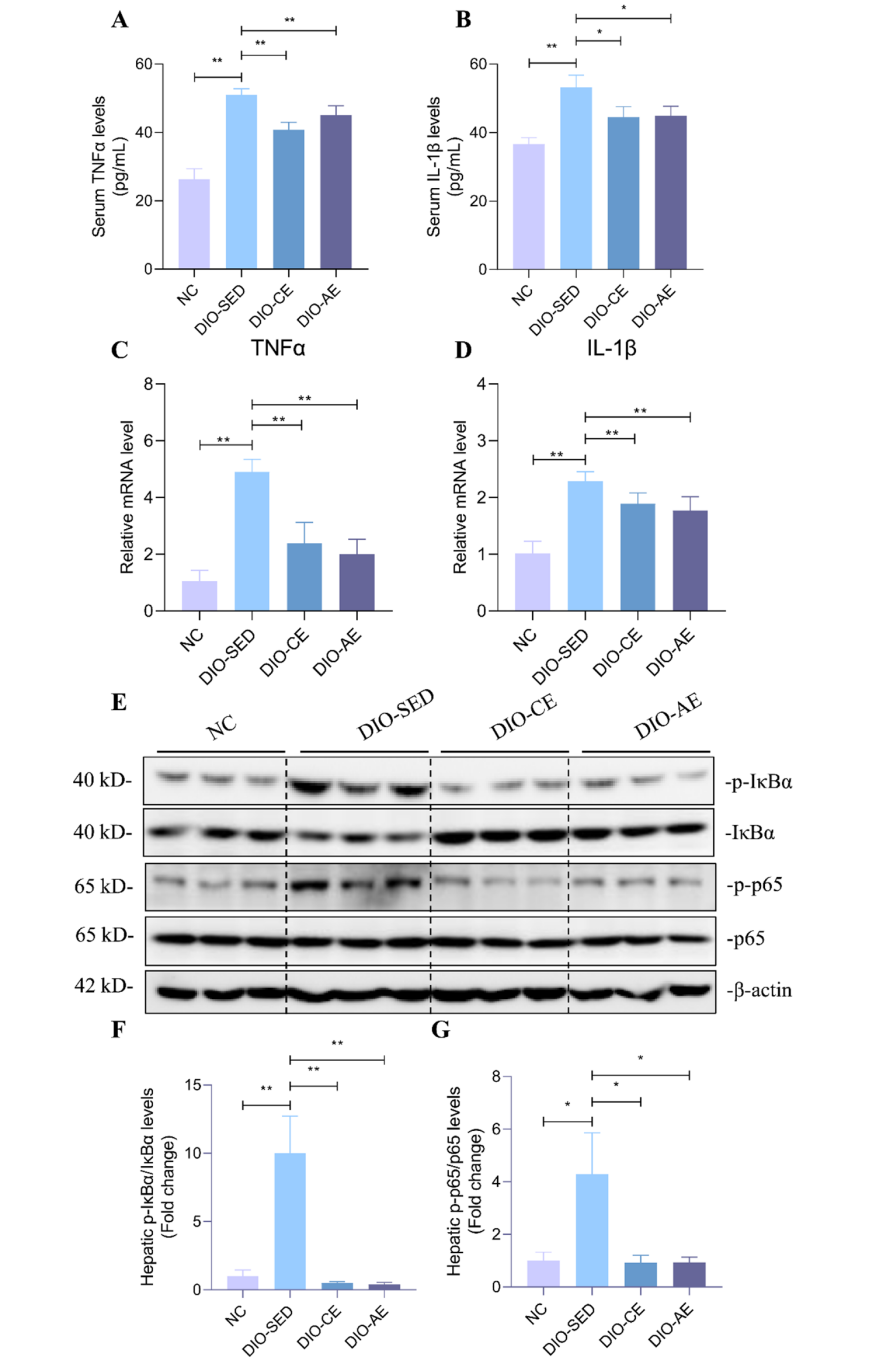
Swimming exercise ameliorates HFD-induced inflammatory responses. **A** The serum levels of TNFα and **B** IL-1β in the indicated groups, **C** the mRNA levels of TNFα and **D** IL-1β in livers of the indicated groups. **E-G** Western blotting of IκBα/P65 in livers of the indicated groups and corresponding quantitative analysis, all experiments were repeated at least 3 times independently. Data is presented as mean ± SD. n = 6. A two-tailed Student’s *t*-test was performed for comparison of results between indicated groups. *p < 0.05, **p < 0.01 between groups

Since NAFLD is closely linked with an inflammatory response, we conducted further research to examine the impact of swimming exercise on the HFD-induced inflammatory response. The results showed that the serum levels of TNF-α and IL-1β were significantly higher in DIO-SED mice than in NC mice. Consistently, the mRNA levels of TNF-α and IL-1β were higher in liver of DIO-SED mice than in NC mice (Fig. 4A-D). Furthermore, serum levels of TNF-α and IL-1β were significantly lower in DIO-CE and DIO-AE mice than in DIO-SED mice. And the mRNA levels of TNF-α and IL-1β were lower in liver of DIO-CE and DIO-AE mice than in DIO-SED mice (Fig. 4A-D). We also examined the impact of swimming exercise on the NF-κB signaling pathway, which is known to be involved in inflammation. Our findings revealed that protein levels of p-IκBα and p-p65 were higher in the livers of DIO-SED mice compared to NC mice (Fig. 4E-G). However, we observed a decrease in the protein levels of p-IκBα and p-p65 in the livers of DIO-CE and DIO-AE mice as compared to DIO-SED mice (Fig. 4E-G). These results indicate that swimming exercise can effectively alleviate hepatic steatosis and inflammation caused by HFD in mice.

### Swimming exercise negatively regulates PPARγ transcriptional network in liver of HFD-induced NAFLD mice

**Fig. 5:**
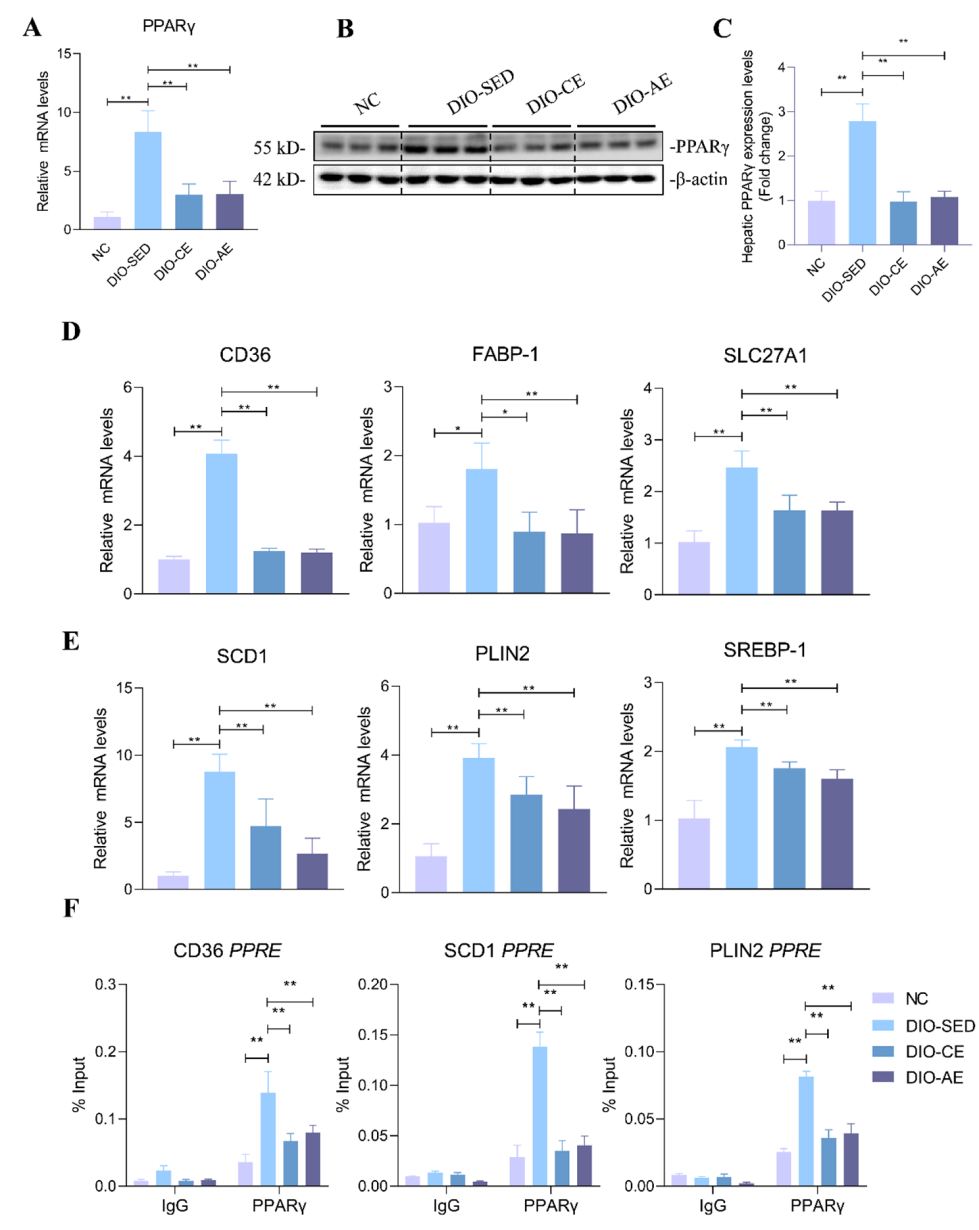
Swimming exercise inhibits PPARγ signaling and recruitment to PPARγ target gene promoters. **A** The mRNA level and **B-C** protein levels of PPARγ in liver of the indicated groups and corresponding quantitative analysis, all experiments were repeated at least 3 times independently. **D** the mRNA levels of PPARγ target genes related to fatty acid uptake and synthesis in liver samples from the indicated group mice. **E** ChIP assays were performed on the CD36, SCD1 and PLIN2 promoters in liver extracted from the indicated group mice (n = 3 per group). Promoter occupancy by PPARγ was analyzed. Data is presented as mean ± SD. n = 6. A two-tailed Student’s *t*-test was performed for comparison of results between indicated groups. *p < 0.05, **p < 0.01 between groups

The beneficial effects of swimming exercise on hepatic steatosis have motivated us to explore the underlying mechanisms. Given that hepatic PPARγ acts as a transcription factor that promotes *de novo* lipogenesis, and increases hepatic triglyceride accumulation, our objective is to examine whether exercise intervention modulates the activity of PPARγ during the progression of NAFLD. We detected the mRNA and protein levels of PPARγ in liver, and the results showed that the mRNA and protein levels of PPARγ were increased in liver of DIO-SED mice compared with NC mice, while these were decreased in liver of DIO-CE and DIO-AE mice than DIO-SED mice (Fig. 5A-C). Furthermore, we detected the mRNA levels of PPARγ target genes involved in lipid metabolism, containing fatty acid uptake-related genes (CD36, FABP-1 and SLC27A1), and fatty acid synthesis-related genes (SCD1, PLIN2 and SREBP-1). The results showed that the mRNA levels of these genes were significantly increased in liver of DIO-SED mice than NC mice, while that were decreased in liver of DIO-CE and DIO-AE mice than DIO-SED mice (Fig. 5D, E). Furthermore, we detected PPARγ recruitment on the CD36, SCD1 and PLIN2 promoters in the liver of mice via ChIP assay. Consistent with gene expression, ChIP assay revealed that PPARγ recruitment on its target promoters was increased in DIO-SED mice than NC mice, while the recruitment of PPARγ on the target promoters were decreased in DIO-CE and DIO-AE mice than DIO-SED mice (Fig. 5F). Our findings suggest that exercise intervention partly suppresses lipid accumulation in NAFLD by downregulating PPARγ-mediated pathways.

PPARα is a transcriptional factor that acts a key role in hepatic lipid beta-oxidation and shares similar response elements with PPARγ on the target promoters. To determine whether exercise intervention also mediates PPARα transcriptional network in the liver, we examined the expression of PPARα and its target genes, including CPT-1, Acox-1, and UCP-2. The results showed that the mRNA levels of these genes were significantly decreased in liver of DIO-SED mice than NC mice, while that were increased in liver of DIO-CE and DIO-AE mice than DIO-SED mice (Supplementary Fig. 1A-D), which is consistent with the results of previous studies (Kim et al. 2013).

### Swimming exercise up-regulated inhibitors of PPARγ transcription in liver of HFD-induced NAFLD mice

**Fig. 6:**
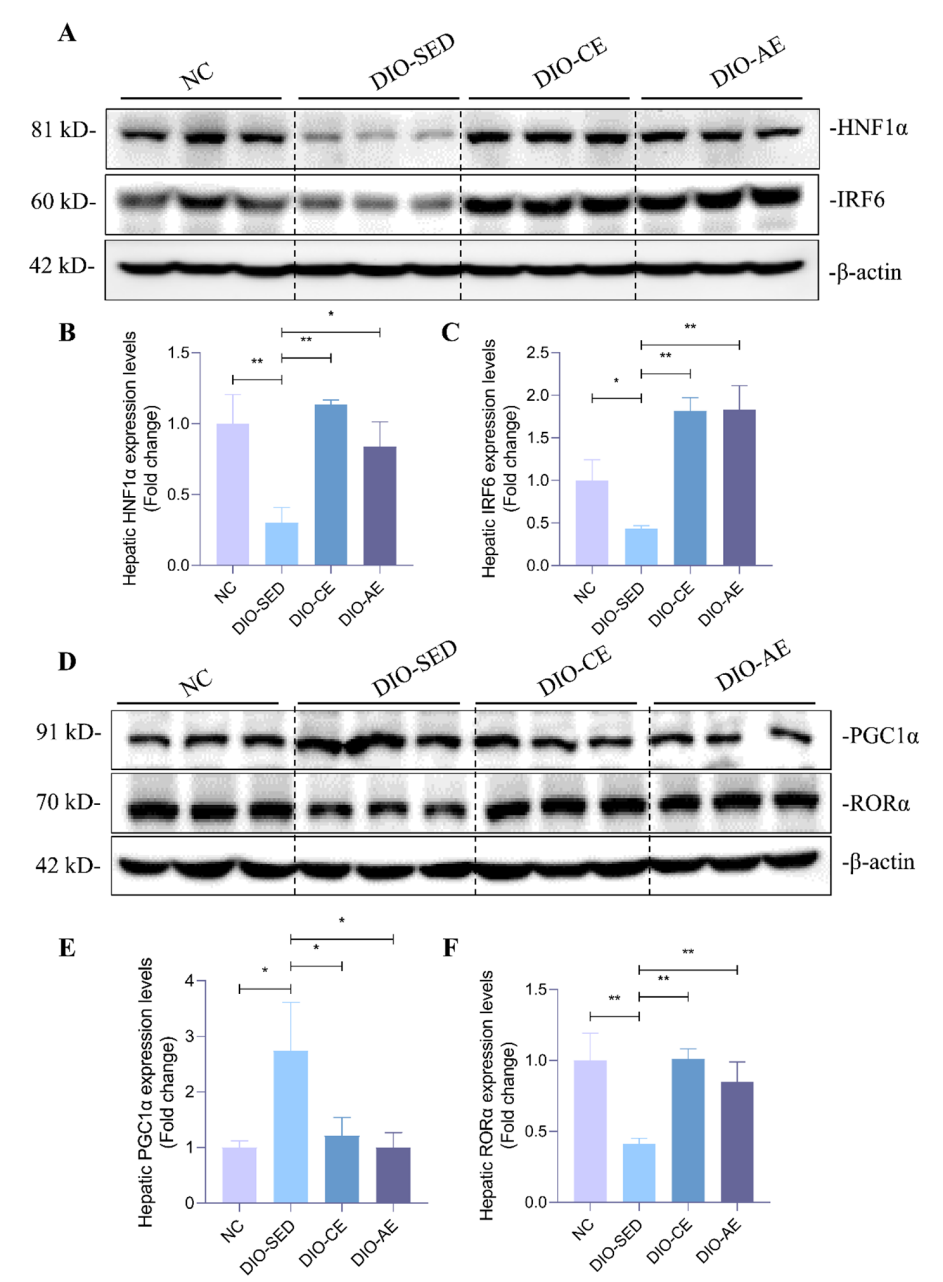
Swimming exercise modulates the expression of PPARγ regulators responsible for its transcription and transcriptional activity. **A-C** Western blotting of HNF1α and IRF6 in livers of the indicated group mice and corresponding quantitative analysis. **D-F** Western blotting of PGC1α and RORα in livers of the indicated group mice and corresponding quantitative analysis, all experiments were repeated at least 3 times independently. Data is presented as mean ± SD. n = 6. A two-tailed Student’s *t*-test was performed for comparison of results between indicated groups. *p < 0.05, **p < 0.01 between groups

Considering that exercise intervention had a regulatory effect on PPARγ and its target genes, we further detected the protein levels of HNF1A, IRF6, which could regulate PPARγ expression at transcriptional level. The western blotting results showed that HNF1A and IRF6 protein levels were decreased in liver of DIO-SED mice compared with NC mice, while these three proteins expression were significantly increased in liver of DIO-CE and DIO-AE mice (Fig. 6A-C). These results indicate that exercise intervention decreased PPARγ and its target genes expression might via regulating transcription inhibitors of PPARγ.

### Swimming exercise modulates regulators of PPARγ transcriptional activity in liver of HFD-induced NAFLD mice

Since PGC1α is the well-known coactivator for PPARγ functions as transcription factor, and RORα negatively regulated transcriptional activity of PPARγ, we detected these two factors protein levels in liver of mice. The results showed that HFD-induced significantly increased PGC1α expression in liver of DIO-SED mice than NC mice, while the PGC1α protein levels were significantly decreased in liver of DIO-CE and DIO-AE mice than DIO-SED mice (Fig. 6D, E). Moreover, HFD-induced significantly decreased RORα expression in liver of DIO-SED mice than NC mice, while the RORα protein levels were significantly increased in liver of DIO-CE and DIO-AE mice than DIO-SED mice (Fig. 6D, F). the These results proposed that exercise intervention might suppress PPARγ transcriptional activity via down-regulating coactivators of PPARγ.

## Discussion

Physical exercise has been demonstrated to be an effective therapeutic approach for alleviating insulin resistance in humans and rodents(Frøsig et al. 2007). Previous research demonstrated that exercise induces the adipose expression of FGF receptor-1 (FGFR1) and β-Klotho (KLB) via peroxisome proliferator-activated receptor-gamma-mediated transcriptional activation in adipose tissues, which in turn sends humoral signals to coordinate multi-organ crosstalk for maintaining metabolic homeostasis(Geng et al. 2019). And exercise could reverse the increased expression of inflammatory cytokines in adipose tissue, reverse adipose tissue inflammation and insulin resistance in diet-induced obese mice(Bradley et al. 2008). Ladder climbing exercise (LC) also increased the phosphorylation of Akt^Ser473^ and AMPK^Thr172^ and reduced TNF-α and IL1-β contents in the quadriceps muscles of HFD-fed mice. Additionally, LC reduced the gene expression of inflammatory markers and attenuated HFD-induced NADPH oxidase subunit gp91phox in skeletal muscles(Effting and Thirupathi 2022).

The liver is the pivotal organ, which is responsible for maintaining glucose homeostasis by balancing glycogenesis and gluconeogenesis in the body. Excess free fatty acid from adipose tissue lipodieresis is released into the blood stream and is absorbed by liver, which accelerates the development of IR(Frühbeck et al. 2014; Sears and Perry 2015). To explore the specific mechanism by which exercise prevents the accumulation of fat in the liver, we detected the expression of PPARγ and its transcriptional network in liver of mice.

Insulin resistance, inflammation, and hepatic steatosis are interconnected phenomena that worsen the progression of NAFLD. Insulin signal transduction and NF-κB pathway represents a significant pathological connection between these metabolic disorders (Dong et al. 2020). Our primary focus was to assess insulin resistance status as well as physiological and metabolic parameters in mice. Continuous HFD feeding significantly increased BW and VFM in mice, and the DIO-SED mice developed severe IR, as proved by higher FBG, FINS and HOMA-IR index. Furthermore, the prolonged consumption of HFD resulted in the activation of the IRS1-Akt pathway, which subsequently triggered the expression of PEPCK and G6Pase enzymes. This led to an increase in gluconeogenesis and facilitated hepatic lipogenesis (Leavens and Birnbaum 2011; Yan et al. 2017). Through the implementation of a swimming exercise intervention, we have shown that swimming can effectively improve insulin sensitivity and glucose tolerance, as well as enhance insulin signaling. Especially, DIO-AE can suppress p-IRS1 expression much more than the DIO-CE group, which may be due to the stronger inhibitory effect of acute exercise on the obese-induced activation of JNK, and consequently resulting in a stronger inhibitory effect on IRS-1 ser307 phosphorylation, as we demonstrated in our previous research (Zhang et al. 2019).

The current study found that HFD treatment resulted in an increase in inflammatory responses, as evidenced by elevated production of pro-inflammatory cytokines in both serum and liver, which was mediated by the activation of NF-κB signaling pathway. Insulin resistance and the inflammatory response can create a harmful cycle, which results in the accumulation of lipids in the liver(Dong et al. 2020). PPARγ is a member of the nuclear hormone receptor superfamily, which plays a pivotal role in the regulation of glucose homeostasis, inflammation, and adipogenesis(Tong et al. 2019). Multiple studies have demonstrated that the hepatic PPARγ gene serves as a promoter of steatosis by initiating the process of *de novo* lipogenesis. Dysfunctional PPARγ signaling leads to unregulated lipogenesis, which is a major contributor to the development of hepatic steatosis and obesity induced by a high-fat diet (HFD)(Zhou et al. 2012; Newberry et al. 2003). Although both short and long-term administration of thiazolidinedione (TZD) and PPARγ agonists, Rosiglitazone, have been demonstrated to alleviate HS in patients with NASH(Stefanovic-Racic et al. 2008; Ratziu et al. 2008, 2010). This contradiction may suggest that TZD treatment alleviates HS due to its primary insulin-sensitizing effect on adipose tissues surpass its steatogenic effects in the liver (Bian et al. 2018; Saraf et al. 2012). Thus, specifically inhibiting hepatic PPARγ is a potential strategy for NAFLD therapy. Previous research revealed that, CAY10566 (an SCD1-specific inhibitor)-treated HFD-fed mice exhibited significantly decreased hepatic steatosis and hepatic lipid droplet accumulation, as well as enhanced AMPK activity and lipophagy (Zhou et al. 2020). And treatment of HFD-fed mice with the SCD1 inhibitor A939572 prevents the diet-induced reduction of hepatic FAAH activity, normalizes hepatic AEA levels, and improves insulin sensitivity (Liu et al. 2013). Our current study reveals that PPARγ signaling is markedly activated in HFD-fed mice, but exercise intervention can reduce PPARγ signaling, which decreased its target genes (CD36, SCD1, PLIN2 etc.) expression, thereby protecting against diet-induced hepatic fatty acid uptake and lipid droplet accumulation in vivo. In general, PPARγ in kupffer cells inhibits inflammation and fibrosis in the liver (Luo et al. 2017), while PPARγ in hepatic parenchymal cell functions as a steatogenic-inducer gene that activates *de novo* lipogenesis and increases hepatic triglyceride accumulation (Zhou et al. 2012; Newberry et al. 2003). Swimming exercise is more likely to inhibit the expression and transcriptional activity of PPARγ in hepatocytes, thereby reducing the liver damage caused by lipid deposition, and thus alleviating the inflammatory response. These findings establish a direct molecular connection between exercise and PPARγ signaling.

Previous studies have revealed that hepatic HNF1A, IRF6 and RORα have the ability to suppress the expression or transcriptional activity of PPARγ, subsequently repressing the lipogenic genes regulated by PPARγ(Kim et al. 2017; Tong et al. 2019; Patitucci et al. 2017). This regulatory mechanism protects against diet-induced hepatic steatosis and obesity. Therefore, the disrupted regulatory mechanism of PPARγ signaling is the primary cause of insulin resistance and glucose intolerance. Our data indicate that exercise intervention might suppress PPARγ signaling via its regulatory factors to control hepatic lipid and glucose metabolism.

Numerous studies have demonstrated that a moderate reduction in weight can significantly improve insulin sensitivity in various tissues and organs, leading to a reduction in insulin resistance and NAFLD (Yu et al. 2016; Younossi et al. 2021; Wilson et al. 2018). The study yielded noteworthy findings, indicating substantial weight loss in DIO-CE mice after undergoing 8 weeks of exercise training. This outcome led to the query of whether the enhancement in IR and NAFLD was a direct result of exercise or if it was solely due to the weight loss. To address this concern, a one-time acute exercise training was implemented, which did not result in any significant alterations in BW or VFW. These results indicate that acute exercise also ameliorates insulin resistance, inflammation response and hepatic steatosis induced by HFD, and modulates PPARγ signaling to preserve lipid homeostasis in the liver. Especially, although acute exercise had no significant effect on LW and LW/BW ratio, it slightly decreased the triglyceride content in liver of mice, which may be due to a stronger enhancement effect of acute exercise on PPARα-mediated fatty acid β-oxidation (Kim et al. 2013).

Generally, our findings demonstrate that both long-term and short-term exercise training effectively modulated hepatic PPARγ signaling and lipid/glucose metabolism. These results establish a clear connection between physical activity and the regulation of hepatic fatty acid and glucose metabolism.

### Electronic supplementary material

Below is the link to the electronic supplementary material.

**Supplementary Material 1**: Primers lists and primary antibody used in current research

**Supplementary Material 2**: Fig. 1 swimming exercise enhanced PPARα and target genes expression. A The mRNA level of PPARα and B-D its target genes in liver samples from the indicated group mice. Data is presented as mean ± SD. n = 6. A two-tailed Student’s *t*-test was performed for comparison of results between indicated groups. *p < 0.05, **p < 0.01 between groups.

## Data Availability

The original contributions presented in the study are included in the article. Further inquiries can be directed to the corresponding authors.
