# Supplementary material for: Swimming exercise ameliorates insulin resistance and nonalcoholic fatty liver by negatively regulating PPARγ transcriptional network in mice fed high fat diet
Source: Mol Med. 2023 Oct 31;29:150. doi: 10.1186/s10020-023-00740-4 (PMC10617119; doi:10.1186/s10020-023-00740-4)
Supplement: Supplementary file 2 — Supplementary Material 2: Fig. 1 swimming exercise enhanced PPARα and target genes expression. A The mRNA level of PPARα and B-D its target genes in liver samples from the indicated group mice. Data is presented as mean ± SD. n = 6. A two-tailed Student’s t-test was performed for comparison of results between indicated groups. *p < 0.05, **p < 0.01 between groups. [file 10020_2023_740_MOESM2_ESM.docx]

**
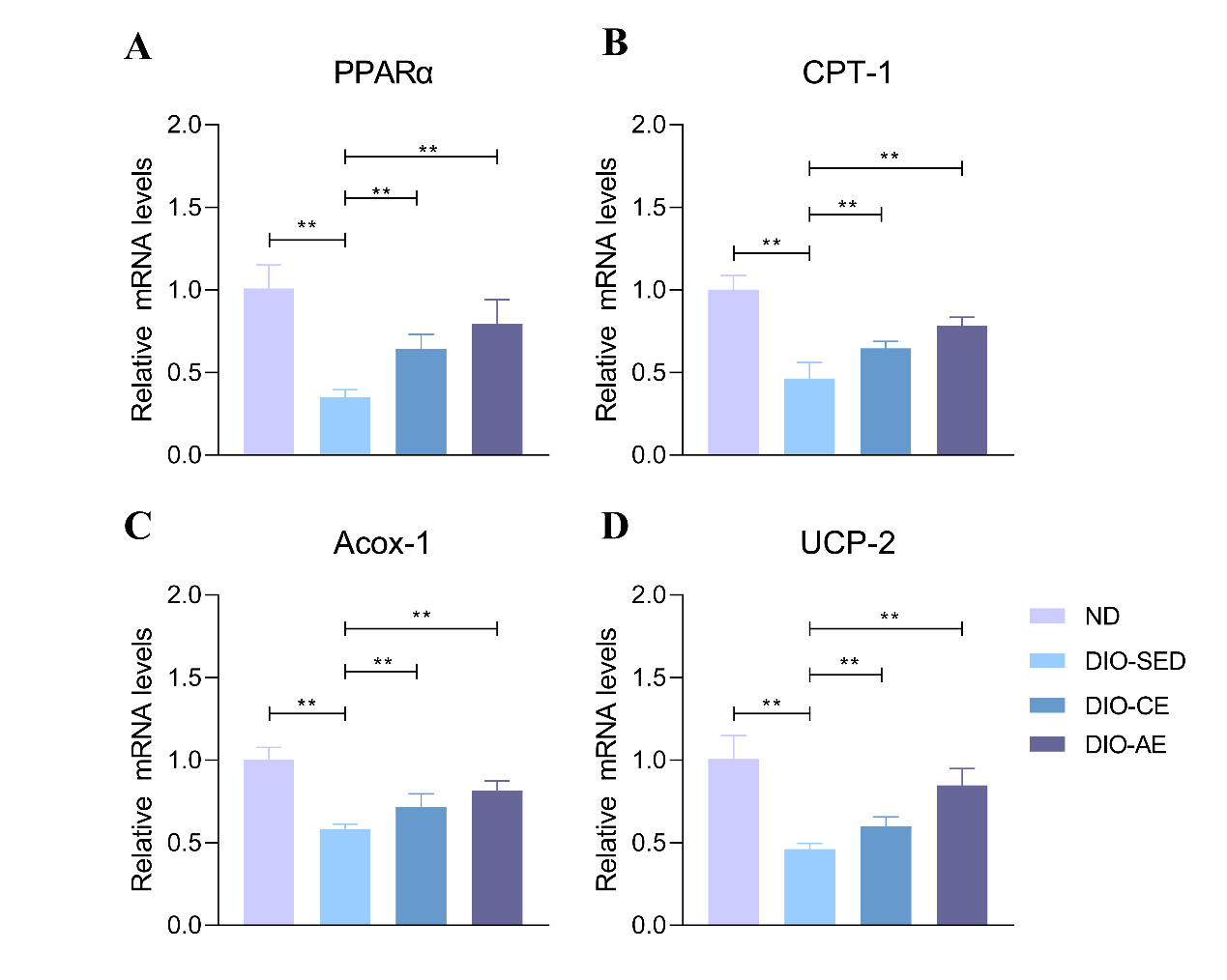
**

**Supplementary Fig. 1 Swimming exercise enhanced PPARα and target genes expression. A** The mRNA level of PPARα and **B-D** its target genes in liver samples from the indicated group mice. Data is presented as mean ± SD. n=6. A two-tailed Student’s *t*-test was performed for comparison of results between indicated groups. *p<0.05, **p<0.01 between groups.
